# Unraveling Sugar Binding Modes to DC-SIGN by Employing Fluorinated Carbohydrates

**DOI:** 10.3390/molecules24122337

**Published:** 2019-06-25

**Authors:** J. Daniel Martínez, Pablo Valverde, Sandra Delgado, Cecilia Romanò, Bruno Linclau, Niels C. Reichardt, Stefan Oscarson, Ana Ardá, Jesús Jiménez-Barbero, F. Javier Cañada

**Affiliations:** 1 CIC bioGUNE, Bizkaia Technology Park, Building 800, 48160 Derio, Bizkaia, Spain; jmartinez@cicbiogune.es (J.D.M.); pvalverde@cicbiogune.es (P.V.); sdelgado@cicbiogune.es (S.D.); aarda@cicbiogune.es (A.A.); 2 Centre for Synthesis and Chemical Biology, University College Dublin, Belfield, Dublin 4, Ireland; ceroma@kemi.dtu.dk (C.R.); stefan.oscarson@ucd.ie (S.O.); 3 School of Chemistry, University of Southampton, Highfield, Southampton SO17 1BJ, UK; bruno.linclau@soton.ac.uk; 4 CIC biomaGUNE, Paseo Miramon 182, 20009 San Sebastián, Gipuzkoa, Spain; nreichardt@cicbiomagune.es; 5 CIBER-BBN, Paseo Miramon 182, 20009 San Sebastián, Gipuzkoa, Spain; 6 Ikerbasque, Basque Foundation for Science, Maria Diaz de Haro 3, 48013 Bilbao, Bizkaia, Spain; 7 Department of Organic Chemistry II, Faculty of Science and Technology, EHU-UPV, 48160 Leioa, Spain; 8 Centro de Investigaciones Biológicas-CSIC, Ramiro de Maeztu 9, 28040 Madrid, Spain

**Keywords:** NMR, molecular recognition, lectin, glycomimetics, DC-SIGN, C-type lectins, fluorinated sugars, ^19^F-NMR, screening, molecular dynamics

## Abstract

A fluorine nuclear magnetic resonance (^19^F-NMR)-based method is employed to assess the binding preferences and interaction details of a library of synthetic fluorinated monosaccharides towards dendritic cell-specific intercellular adhesion molecule 3-grabbing non-integrin (DC-SIGN), a lectin of biomedical interest, which is involved in different viral infections, including HIV and Ebola, and is able to recognize a variety of self- and non-self-glycans. The strategy employed allows not only screening of a mixture of compounds, but also obtaining valuable information on the specific sugar–protein interactions. The analysis of the data demonstrates that monosaccharides Fuc, Man, Glc, and Gal are able to bind DC-SIGN, although with decreasing affinity. Moreover, a new binding mode between Man moieties and DC-SIGN, which might have biological implications, is also detected for the first time. The combination of the ^19^F with standard proton saturation transfer difference (^1^H-STD-NMR) data, assisted by molecular dynamics (MD) simulations, permits us to successfully define this new binding epitope, where Man coordinates a Ca^2+^ ion of the lectin carbohydrate recognition domain (CRD) through the axial OH-2 and equatorial OH-3 groups, thus mimicking the Fuc/DC-SIGN binding architecture.

## 1. Introduction

Carbohydrates are ubiquitous in nature in different combinations, from single monosaccharides to extremely complex glycoconjugates. The possibility of finding different molecular structures, by variation of the stereochemistry of hydroxyl groups and the regiochemistry of the glycosidic linkages, grows geometrically as compared with other linear biopolymers. Ligand-based NMR experiments are widely used to study the interactions between carbohydrates and receptors in solution [1,2,3,4]. Among these methods that are especially powerful for screening purposes, ^19^F-based approaches present a number of important advantages [5,6,7,8]. In this study, we applied a strategy that allows the screening of a library of synthetic fluorinated monosaccharides to study the interactions with the biologically relevant lectin dendritic cell-specific intercellular adhesion molecule 3-grabbing non-integrin (DC-SIGN) [9] and, at the same time, obtain ligand-specific chemical mapping information.

DC-SIGN is a C-type lectin expressed by dendritic cells [10]. It acts as an adhesion receptor in cell–cell interactions, and plays crucial roles in the DC migration and adaptive immune response initiation [10]. It is extensively reported that DC-SIGN is able to bind the HIV-1 envelope glycoprotein, gp 120, which is exploited by the virus to enhance its infectivity of T cells [11,12]. The importance of DC-SIGN is, therefore, evident and a full understanding of the recognized mechanism and its different ligands remains a matter of interest. Glycan binding to DC-SIGN occurs through direct coordination with one of the structural Ca^2+^ ions of the lectin carbohydrate recognition domain (CRD) and the additional contacts with the surrounding amino acid residues define the sugar specificity. An evaluation of the monosaccharide binding affinity revealed Fuc as the preferred ligand, followed by Man (two-fold weaker) [13]. However, the binding specificity of DC-SIGN is remarkably broad, and its glycan binding promiscuity has been related to its different biological roles [14].

In this study, the sugar recognition profile of DC-SIGN is studied in detail. Employing the screening and ligand chemical mapping protocol with a rationally designed mixture of 26 monofluorinated monosaccharides, we confirm the previously reported preference for Fuc and Man moieties exhibited by DC-SIGN. Interestingly, evidence of the interaction between Gal moieties and the lectin is also found, as well as the existence of a new binding epitope for Man residues that involves direct contacts of Man O2/O3 with the Ca^2+^ of DC-SIGN CRD, which was not previously detected.

## 2. Results and Discussion

### 2.1. ^19^F-NMR-Based Chemical Mapping

**Figure 1:**
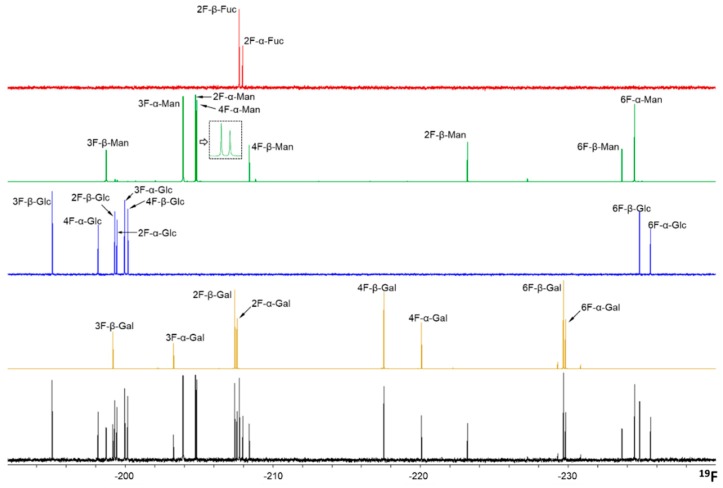
^19^F-NMR proton decoupled spectra of the monofluorinated monosaccharides grouped by sugar type: F-Gal (yellow), F-Glc (blue), F-Man (green), and F-Fuc (red). The mixture of all fluorine-containing monosaccharides employed herein is shown in black at the bottom. A close-up of the 2-F-α-Man and 4-F-α-Man signals is indicated for clarity. The concentration of the molecules was 0.8 mM ([α] + [β]). A total of 16 scans were acquired, with a repetition time of 3 s.

The wide range of chemical shifts displayed by the ^19^F nucleus permits the identification of every fluorine-containing monosaccharide within the complete mixture in a straightforward manner, using a standard proton decoupled ^19^F spectra. Both α- and β-anomers are perfectly distinguished for every sugar. Therefore, the spectrum of the mixture contains 26 different ^19^F-NMR signals (13 distinct fluorine-containing monosaccharides and two anomers of each, Figure 1).

**Figure 2:**
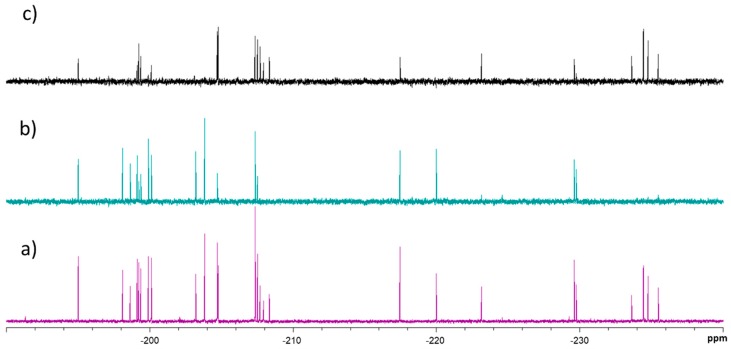
^19^F-NMR relaxation filter experiments performed for the fluorine-containing monosaccharide library in the presence of tetrameric dendritic cell-specific intercellular adhesion molecule 3-grabbing non-integrin (DC-SIGN). The F-sugar/lectin ratio was 47:1. (**a**) Spectrum acquired without T_2_ relaxation filter, (**b**) spectrum acquired after 2.4 s of T_2_ relaxation filter, and (**c**) difference spectrum. Only the peaks of those molecules that display a significant decrease in their T_2_ are present. These peaks correspond to DC-SIGN binders.

First, the transverse relaxation times (T_2_) of the corresponding ^19^F nuclei were quantitatively estimated using the conventional Carr-Purcell-Meiboom-Gill (CPMG) spin echo pulse sequence [15] as a reference for the following analysis (Table S1 in Supporting Information). The screening is based on monitoring changes in the transversal relaxation times of the fluoro-monosaccharides, before (T_2,free_) and after (T_2,obs_) addition of the lectin, which are indicative of binding. Although visual comparison of the signals in the absence and presence of the lectin for a specific relaxation filter time, or the observation of the time evolution of the signals (Figure 2), allows a rapid hit identification, it is considerably more reliable to calculate the complete relaxation curves to estimate T_2,obs_ of the ^19^F nuclei in the mixture.

**Figure 3:**
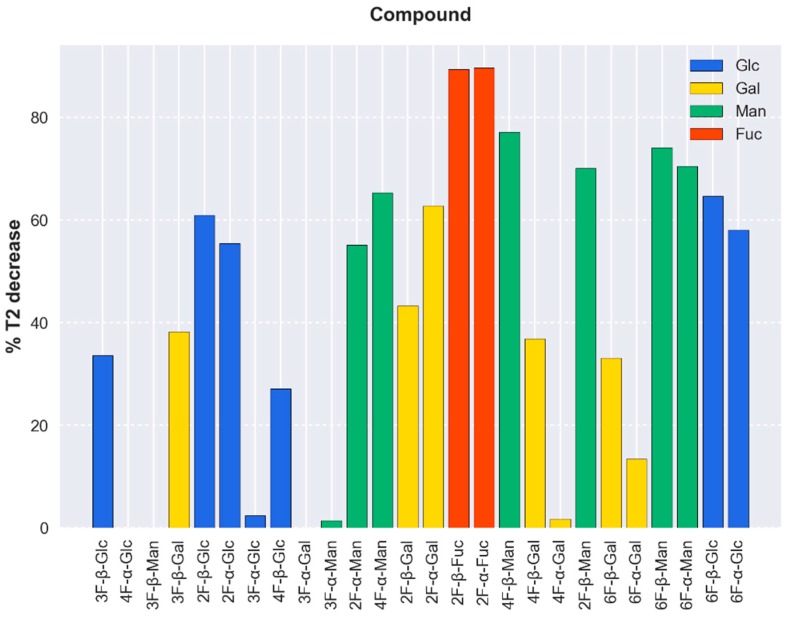
Percentage of decrease of the T_2_ values between the free and bound states of the fluorine-containing monosaccharide library obtained from the ^19^F Carr-Purcell-Meiboom-Gill (CPMG) experiments. In this particular case, the ligand/DC-SIGN ratio was 47:1. Binders of the lectin display a remarkable decrease in the normalized T_2_ values, above 40%. Qualitatively, very weak or non-binders show decreases below 40%. The order in the x-axis corresponds to the ^19^F chemical shift. The molecule whose ^19^F signal displays the lowest field chemical shift is at the left, while that whose ^19^F signal is at the highest field chemical is at the right.

Thus, the interaction of tetrameric full-length DC-SIGN with the monosaccharide library was evaluated by computing the decrease of the T_2_ values of the fluorinated molecules (Figure 3) in the presence of a given amount of the lectin, normalized for each peak with respect to its T_2,free_ [8]:

(1) $$\%\;T_{2,decrease}=\frac{T_{2,free}-T_{2,obs}}{T_{2,free}}\times 100$$

**Figure 4:**
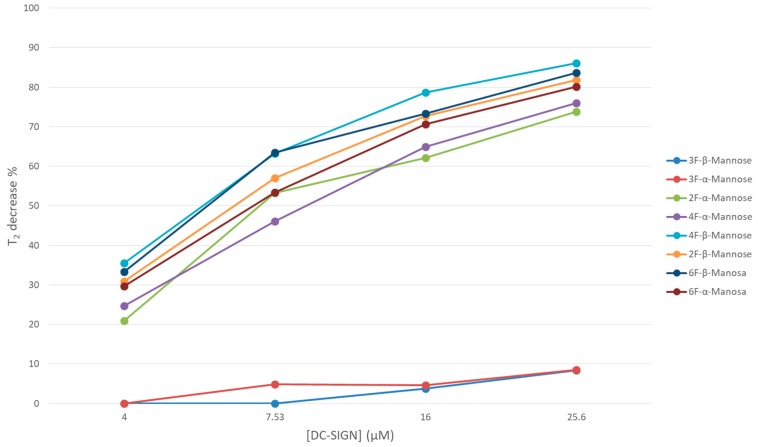
Percentage of decrease of the T_2_ values for different ligand/lectin molar ratios deduced for the fluorine-containing Man moieties, as obtained from the ^19^F-NMR relaxation filter experiments.

In particular, controlled amounts of DC-SIGN concentrations were added to the fluorinated sugar library and the observed T_2_ values were deduced. The analysis of the data permitted deducing a systematic reduction of the signal intensities in specific cases (Figure 4), which were correlated with the presence of binding. Interestingly, even rather weak interactions of the molecules with the lectin were detected. Very few signals, corresponding to 3-, 4-, and 6-F-Gal, 3-F-Man, and 3- and 4-F-Glc, were very poorly affected by the presence of DC-SIGN (T_2_ decay below 40%). The most perturbed signals corresponded to both anomers of 2-F-Fuc (about 90%), followed by Man moieties with fluorine substitution at positions 2, 4, and 6 (60–80%). As well, 2-, 6-F-Glc, and 2-F-Gal underwent a measurable reduction in their T_2_ (40% to 60% of decay). Fittingly, these observations are in good agreement with previous reports on the natural nonfluorinated sugars [13]. The obtained data also indicate that the anomeric OH does not seem to be involved in the sugar recognition by the lectin. It is evident that the sensitivity of this NMR protocol is fairly high, and that even very low mM affinities binders (such as Gal (Kd 72 mM), a five-fold weaker binder than Man) is also detected [13]. Indeed, the architecture of the CRD of DC-SIGN allows a rather shallow and solvent exposed sugar-binding site, which is fairly accessible to poor affinity ligands.

From the structural perspective, it was shown that Fuc, Man, and even GlcNAc in different oligosaccharides are able to coordinate the calcium ion of DC-SIGN CRD through their OH-3 and OH-4 diols, which also establishes key hydrogen bonds with the neighboring amino acid residues [16,17]. Other C-type lectins also interact with sugars through these hydroxyl groups (i.e., LSECtin and Langerin with Fuc, Man, and GlcNAc) [18,19]. In fact, it has been described that the Man versus Gal specificity in C-lectins is dictated by a short amino acid sequence at the lectin binding site [20]. Therefore, given the subtle interaction network of the coordinating OH, it was expected that replacement of any of these OH-3 and OH-4 hydroxyl groups by a fluorine atom would preclude the sugar-lectin recognition.

As anticipated, the chemical mapping analysis reveals the involvement of Man OH-3, Glc OH-3, and Glc OH-4 as crucial elements in the binding event, but surprisingly not Man OH-4. It also highlights the absence of strong interactions between most Gal moieties and DC-SIGN, with the exception of 2-F-Gal, which could coordinate the calcium ion of the lectin through OH-3 and OH-4. A similar binding mode has been described for Langerin, another C-Type lectin closely related to DC-SIGN [21]. The fact that 6-F-Gal barely interacts with the receptor, despite having the OH-3/OH-4 groups available, suggests some participation of the OH-6 in the very weak binding of the Gal residues [13]. To our knowledge, this is the first evidence of the interaction between Gal and DC-SIGN through OH-3/OH-4-Ca^2+^ contact, disregarding the reported nonphysiological binding detected for Gal with C-type mannose-binding proteins via coordination of Ca^2+^ with OH-1 and OH-2 [13,22]. It is worth mentioning that the residual binding observed for the molecules exhibiting a T_2_ decrease below 40% (i.e., for those species considered here as very weak or non-binders), might be related with such a nonphysiological interaction. Interestingly, an anomeric preference for the beta configuration is deduced for those fluorinated-Gal and Glc entities that are scarcely affected by the presence of the lectin, namely 3-, 4-, and 6-F-Gal, and 3- and 4-F-Glc. Since this differentiation does not occur for 3-F-Man, whose hydroxyl at position two is in an axial arrangement, it is possible that the OH-1/OH-2 Ca^2+^ interaction requires a fixed equatorial-equatorial disposition of the contiguous hydroxyl groups in order to take place.

Although the relative decrease in T_2_ of the compounds in the mixture by the presence of the lectin depends on several factors, it is related, at least qualitatively, with ligand-receptor affinity as certain conditions are favorable in this particular fluorinated library. Given the small size of the ligands and their similar shape and chemical nature, it is assumed that their T_2_ values in the bound state and association rate constants, k_on_ (probably under diffusion control), will be similar. The T_2_ values in the free state (1.43 ± 0.73 s), as well as the employed ligand concentrations (0.50 ± 0.15 mM), are also comparable. In addition, it is expected for these molecules sharing the same binding spot on the lectin that the chemical shift differences of the ^19^F nucleus between the free and bound states, which dictates the exchange contribution to the observed T_2_ [5], will also be alike (especially for pairs of anomers when the binding does not involve the hydroxyl at C-1 position). Indeed, the obtained results suggest a general affinity tendency that is in agreement with the current knowledge of DC-SIGN sugar preferences. Table 1 shows the average decrease in T_2_ values by monosaccharide type, discarding those that show a T_2_ decrease lower than 40%, herein considered non-binders. Even though only semiquantitative, the fluorine-containing Fuc and Man species experience a more pronounced decrease in their observed T_2_ values, followed by Glc and Gal residues.

In addition to the identification of 2-F-Gal as a DC-SIGN ligand, these NMR experiments also revealed unexpected findings regarding the binding with Man. As mentioned before, the available X-ray structures show that the interaction of oligosaccharides through Man moieties exclusively takes place by the simultaneous contact of the Ca^2+^ at the DC-SIGN binding site with hydroxyls OH-3/OH-4. The results presented above, however, suggest that the OH-4/Ca^2+^ interaction is actually not essential for binding since 4-F-Man moieties are recognized by the lectin. Indeed, all fluorinated Man molecules displayed a marked decrease in their observed T_2_ values upon increasing amounts of tetrameric DC-SIGN in solution, except both 3-F-Man anomers (Figure 4). This fact provides further evidence that the presence of fluorine at position three prevents binding, while the recognition process still takes place when the F substitution occurs at C4. Thus, in the absence of a donor hydroxyl group at position four, other alternative binding modes take place. Based on the sugar recognition requirements observed for DC-SIGN and other C-Type lectins, it can be hypothesized that the alternative binding modes would likely involve the simultaneous contact of hydroxyls OH-2/OH-3 with the Ca^2+^ ion.

**Figure 5:**
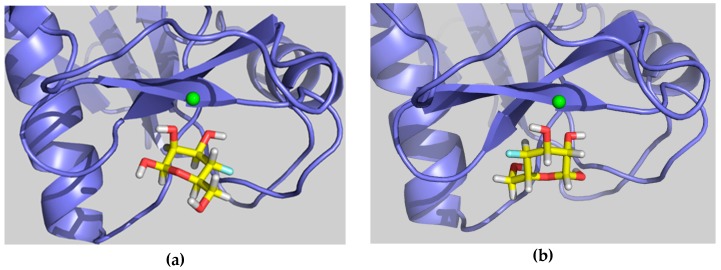
Representative structures of β-4-F-Man binding pose A, mimicking Fuc (**a**) and binding pose B, mimicking Man (**b**).

**Figure 6:**
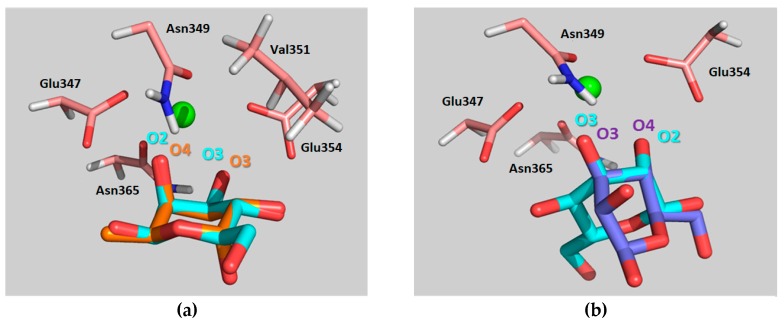
(**a**) Superimposition of Man (blue) in binding pose A and Fuc (orange) as found in PDB code 1SL5 and (**b**) superimposition of Man in binding pose B (light blue) with Man (purple) as found in PDB code 2IT5. Oxygen atoms that coordinate Ca^2+^ are indicated in the same color as their sugar ring. The most relevant residues of the protein stabilizing the sugar at the binding site are shown in each case. All Man hydrogens, as well as the other residues forming the oligosaccharides contained in the original PDB structures have been removed for the sake of clarity.

The feasibility of these alternative binding modes was explored using molecular modeling procedures. In particular, two putative binding poses were initially built. The first one, binding pose A, was generated using the geometry with PDB code 1SL5 (DC-SIGN complexed to lacto-*N*-fucopentaose), placing the 4-F-Man moiety at the Fuc site by superimposing the OH-2/OH-3 groups of 4-F-Man with OH-4/OH-3 of Fuc (see Figure 5a and Figure 6a, with an equivalent Man molecule in this binding pose). The second one, binding pose B, was generated using the geometry with PDB code 2IT5 (DC-SIGN bound to Man6, a high-mannose *N*-glycan, with the sugar arranged in the major observed orientation), placing the 4-F-Man moiety at the calcium coordinating Man site by superimposing the OH-2/OH-3 groups of 4-F-Man with OH-4/OH-3 of Man (see Figure 5b and also Figure 6b, with an equivalent Man molecule in this binding pose). Both proposed poses are related through a 180° rotation around the line that bisects the pyranose ring across the C3–C2 bond and display two contiguous hydroxyl groups attached to the calcium ion.

### 2.2. Molecular Dynamics Simulations

The conformational stability of the complexes was studied by molecular dynamics (MD) simulations in explicit water. To begin with, MD simulations were run for the complexes of DC-SIGN CRD with the natural sugars α-Fuc and α-Man coordinating the principal Ca^2+^, as found in the 1SL5 and 2IT5 X-ray structures (the other residues of the original oligosaccharides were removed). The amino acid sequence numbering employed hereafter starts by one for the first residue, the equivalence with the PDB structure (which starts by 252) is found on Table S1.

The MD simulation showed a stable lectin-Fuc complex throughout the complete trajectory. Apart from the typical coordination of the Ca^2+^ by the equatorial OH3- and the axial OH-4 hydroxyl groups of the α-Fuc pyranose ring, essential sugar-lectin hydrogen bonds are also observed with amino acids that coordinate the Ca^2+^ ion during the simulation. Key binding interactions take place with Glu354, Glu347, Asn365, and Asn349. The α-Fuc OH-3 and OH-4 act both as hydrogen bond donors and acceptors, while OH-2 acts only as a donor. Moreover, stabilizing van der Waals contacts of α-Fuc H-2 with the methylene group of Val351 are observed, as previously proposed by Drickamer et al. [17]. These major interactions throughout the simulation time are summarized in Table S2.

After Fuc, Man is the monosaccharide that displays higher affinity for DC-SIGN, even though the binding is still weak [13]. It has been suggested that the smaller affinity as compared with that of Fuc could arise from the above-mentioned hydrophobic contact between Fuc H-2 and Val351, which is not present in the complex with Man. In all X-ray diffraction structures, the orientation of Man at the binding site allows its equatorial OH-3 and OH-4 groups to coordinate the Ca^2+^ ion. Accordingly, the MD simulation revealed that these groups provide stabilizing contacts with Glu354, Glu347, Asn365, and Asn349, although in an inverted fashion with respect to α-Fuc (Table S3). In contrast, no sugar hydroxyls other than the calcium coordinating groups are involved in binding to the lectin. Remarkably, conversion from the X-ray binding mode (involving Ca^2+^ coordination by OH-3/OH-4), to the proposed pose A (involving Ca^2+^ coordination by OH-2/OH-3) is also observed during the MD simulation (beyond 100 ns of simulation time).

The simulation procedure was then tested for the complex of DC-SIGN CRD with the natural α-Man (nonfluorinated) using the two proposed binding poses involving Ca^2+^ coordination by OH-2/OH-3, A and B, as starting geometries. In pose A, the α-Man pyranose ring is entirely superimposable with that of α-Fuc (PDB 1SL5), fulfilling the same geometric features described above for such. More specifically, the oxygen and C3 atoms of the ring coincide in both sugars, while the other carbon atoms are symmetrically presented in one sugar with respect to the other (specifically C1_Man_-C5_Fuc_ and C2_Man_-C4_Fuc_, Figure 6a). In pose B, the O2-C2-C3-O3 segment of the α-Man pyranose ring is superimposed onto the O4-C4-C3-O3 of Man found on the X-ray crystal structure (PDB 2IT5), which translates into a change in the orientation of the pyranose ring plane (Figure 6b). As mentioned above, binding poses B and A are related through a 180° rotation about the C2–C3 bond bisector (Figure 5).

**Figure 7:**
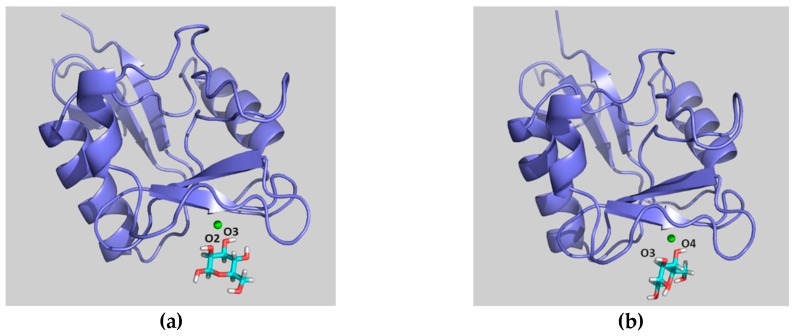

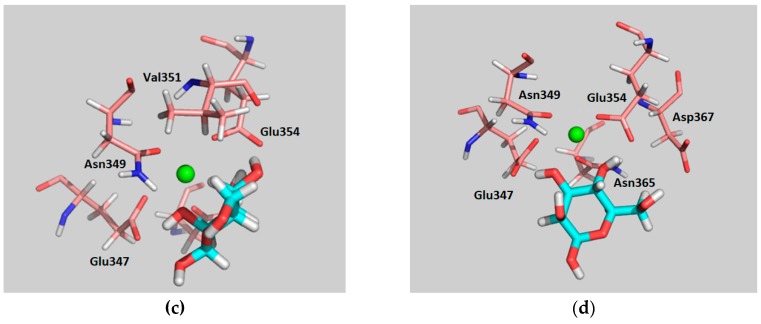
Snapshots from the molecular dynamics (MD) simulation of β-d-Man/DC-SIGN complex using pose A as the starting geometry. The general view is presented at the top panels, while expansions of the binding site are shown at the bottom panels. Panels (**a**) and (**c**) show Man (blue) in binding pose A. Panels (**b**) and (**d**) show Man in the binding pose of the X-ray crystal structures. Both binding modes are found during the MD simulations, regardless of the selected starting pose.

Interestingly, both simulations displayed rather different behaviors. When binding pose A was used as a starting geometry, the α-Man moiety is able to switch its pose along the MD trajectory (after approximately 100 ns), adopting the binding mode described for Man in the available X-ray crystal structure of PDB 2IT5 (Figure 7). In fact, as mentioned above, this interchange between the two binding poses (the one of the crystal structure and binding pose A) takes place regardless of the starting geometry used. Thus, α-Man in binding pose A turns around the O3-Ca^2+^ bound axis, to find the usual binding pose that shows the direct interaction of the equatorial OH-3 and OH-4 hydroxyl groups with the Ca^2+^ ion. Nevertheless, during the first 100 ns of simulation where binding pose A takes place, the observed protein–sugar interactions (Table S4) resemble those described above for α-Fuc, with the corresponding geometry changes (C1_Man_–C5_Fuc_ and C2_Man_–C4_Fuc_, Table S2). Stabilizing hydrogen bonds between Man OH-4 (as donor) and Glu354 are predicted, accompanying those involving α-Man OH-2 and OH-3, as well as hydrophobic contacts between α-Man H-4 and Val351. In particular, α-Man OH-4 only acts as a hydrogen bond donor, while α-Man OH-3 acts as both a donor and an acceptor. In the second part of the simulation, α-Man adopts the presentation described in the X-ray complexes, and O3 and O4 now coordinate the Ca^2+^ ion (Table S5). The analysis of the interactions during the whole simulation is gathered in Table S6 and Figure S1. Obviously, the most populated hydrogen bond contact is that between α-Man OH-4 and Glu354, shared in both binding modes (Figure S2), which takes place for 99% of the time the complex remains bound.

On the other hand, MD simulations for α-Man, using pose B as the starting geometry, showed only this particular binding mode during the whole trajectory (200 ns). The key contacts are described in Table S7. Herein, the participation of α-Man OH-3 in establishing hydrogen bonds is rather minor, in contrast to the observations for the reference X-ray structure. Instead, α-Man OH-4 acts now as a hydrogen bond donor, and there is no hydrophobic stabilization with Val351. Overall, the number of observed interactions for this binding mode is smaller than those for binding mode A.

Once the MD protocol had been satisfactorily evaluated with Fuc and Man, the simulations were conducted for β-4-F-Man bound to DC-SIGN CRD. The two proposed binding poses described above were employed as starting geometries.

For the complex starting from pose A, the analysis of the trajectory revealed similar interactions to those described above for α- Fuc (Table S8), including the stabilizing van der Waals contact with Val351. The major difference in the recognition pattern for both sugars is found at position four of β-4-F-Man (equivalent to position two of α-Fuc). The presence of the fluorine atom prevents the formation of the hydrogen bond with the carboxylate group of Glu354, which was present throughout the simulation time with α-Fuc. The Man OH-2 mainly acts as hydrogen bond donor, with a smaller role as an acceptor, while Man O3 is involved as both a donor and an acceptor (Figure S3). The MD results for this analogue are also similar to those predicted in the first part of the MD simulation carried out for α-Man in this pose. The MD results also suggest that the anomeric position is not directly involved in the recognition process, in agreement with the experimental observations. As expected, the fluorine atom does not seem to play a role on the sugar stabilization at the binding site.

The analysis of the MD simulations carried out with β-4-F-Man in binding pose B showed that the main stabilizing interactions correspond to the hydrogen bond network formed between the Ca^2+^-coordinating O2 and O3 atoms of the 4-F-Man moiety with the side chains of Glu347, Glu354, and Asn365 at the binding site. There is no participation of Asn349, contrary to the simulation with the X-ray geometry and the α-Man structure in binding pose B. In addition, Man OH-3 is now strongly involved as a donor in the hydrogen bond interaction with Glu347, taking the role of OH-4 in the regular sugar (Table S9 and Figure S4). Nevertheless, the orientations of β-4-F-Man and α-Man at the binding site are very similar (Figure S5). Again, there is no van der Waals contact between the sugar and Val351, and neither the fluorine atom nor the OH at the anomeric position, which is solvent exposed, are involved in intermolecular interactions with the lectin.

In summary, the interactions established by α-Man and β-4-F-Man in binding pose A are similar to those found for Fuc in PDB 1SL5. Obviously, for binding pose B, the sugars exhibit a totally different binding mode from that described for Man in PDB 2IT5. The detailed analysis of the MD trajectories suggests that binding pose A is more favorable than pose B, according to the number of stabilizing interactions found for both. Furthermore, the simulations suggests that the fluorinated molecule 4-F-Man is recognized by DC-SIGN with smaller affinity than the natural Man. Additionally, the corresponding poses were tested for a putative complex with α-3-F-Man, but all the trials showed the dissociation of the complex prior to the production dynamics stage, in agreement with the experimental results.

### 2.3. 1H-STD Experiments

**Figure 8:**
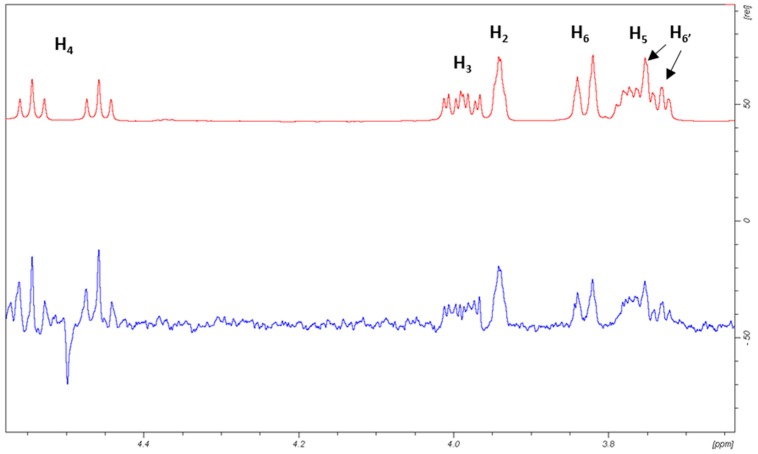
Expansion of the sugar region of the STD-NMR spectrum obtained for α-OMe-4-F-Man (5 mM) in the presence of DC-SIGN (65 μM, ligand/lectin ratio is 77/1). Top: Off-resonance spectrum. Bottom: STD spectrum. H-4 shows the largest STD signal. The spectra were obtained for 1024 scans at 298 K with off-resonance irradiation at 60 ppm. In this particular case, protein irradiation was set at 0.81 ppm with a saturation time of 2 s. Additional experiments were carried out irradiating at the aromatic region and employing different saturation times (0.5, 1.0, and 3.5 s). No STD was obviously observed in the absence of DC-SIGN.

^1^H-STD NMR experiments were then carried out for a mixture of DC-SIGN with α-OMe-4-F-Man to experimentally evaluate the binding epitope. As described above, the ^19^F-NMR relaxation filter experiments showed that the anomeric configuration does not seem to play any important role in the binding event, which was also corroborated by the MD simulations. Anyhow, additional MD simulations were performed for the corresponding A and B poses of the α-OMe-Man/DC-SIGN system to verify once again that this was still the case and that the obtained trajectories were indeed similar to those described above for the free anomer. Fittingly, the use of the α-OMe derivative notably simplifies the ^1^H-NMR spectrum, allowing to satisfactorily assign the STD intensities (Figure 8).

**Figure 9:**
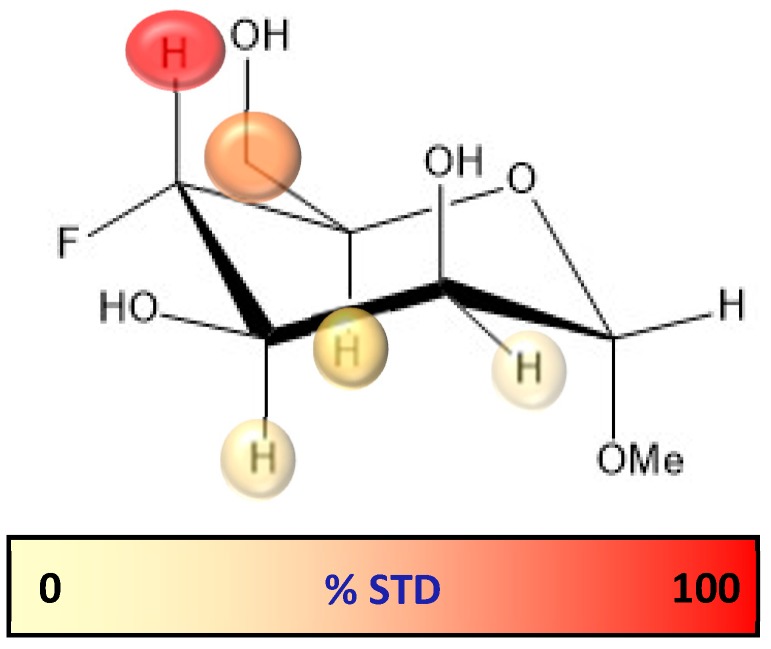
Relative STD profile for α-OMe-4-F-Man (5 mM) in the presence of DC-SIGN (65 uM, ligand/lectin ratio is 77/1), normalized to that of the H-4 proton (100% relative STD).

Upon irradiation at the aliphatic region, H-4 showed the largest STD, followed by H-6 (65% with respect to H-4), while H-3 and H-2 showed the lowest STD responses (40–50%, Figure 9, Table S10 and Figure S6).

**Figure 10:**
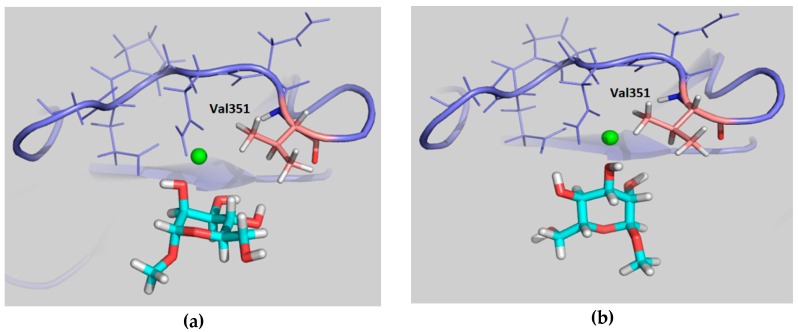
Model frames from the MD simulation performed for α-Me-Man bound to DC-SIGN in poses A (**a**) and B (**b**). For pose A, H-4 and H-6 are close to Val351, whereas H-2 and H-3 points towards the polar region defined by Asn349, Asn365, and Glu354. In contrast, for pose B, the alternative situation takes place, with H-2 and H-3 pointing towards Val351.

The observed large STD at H-4 is satisfactorily explained by pose A, since when using this geometry this proton is in direct contact with Val351 (Figure 10a). Furthermore, the observed STD for H-6 also agrees with this binding mode, as these protons are also close to the aliphatic chain of Val351, whereas H-2 and H-3 are far from the hydrophobic site. On the other hand, the opposite STD profile should be expected for binding mode B, in which H-2 and H-3 are facing Val351 while H-4 and H-6 are far from aliphatic hydrogens, exposed to the solvent (Figure 10b). Therefore, the STD results strongly support the presence of a major contribution of binding pose A with respect to pose B, and confirm the presence of a binding mode for 4-F-Man to DC-SIGN, which involves a direct contact of OH-2 and OH-3 with the Ca^2+^ ion. Regarding the influence of the ^19^F nucleus in the observed STD, it is safely assumed that it is almost negligible. We have extensively explored the ^1^H to ^19^F saturation transfer process [6], which was found rather difficult to detect. In fact, the best way to use ^19^F to detect STDs was to employ the regular ^1^H to ^1^H STD, followed by coherence transfer to ^19^F through the ^2^JFH/^3^JFH scalar coupling constants [6,23].

## 3. Materials and Methods 

### 3.1. Preparation of F-Monosaccharide Library

The library of mono-fluorinated monosaccharides was rationally prepared using the three basic monosaccharide units: d-glucose (Glc), d-galactose (Gal), and d-mannose (Man). The fluorine-by-hydroxyl substitution took place at every position (2-, 3-, 4- and 6-) with the exception of the anomeric site, therefore, ending up with a mixture of α/β-anomers of each fluoro-sugar. Additionally, a l-fucose (Fuc) derivative, 2-deoxy-2-F-fucose, was also included in the library. Most fluorinated monosaccharides were obtained from a commercial source (Sigma-Aldrich, Madrid, Spain; or Carbosynth, Compton, UK): 2-deoxy-2-F-glucose, 3-deoxy-3-F-glucose, 4-deoxy-4-F-glucose, 6-deoxy-6-F-glucose, 2-deoxy-2-F-galactose, 3-deoxy-3-F-galactose, 4-deoxy-4-F-galactose, 6-deoxy-6-F-galactose, 2-deoxy-2-F-mannose, 3-deoxy-3-F-mannose, 4-deoxy-4-F-mannose and 2-deoxy-2-F-fucose. The 6-deoxy-6-F-mannose and α-Methyl-4-deoxy-4-F-mannose, employed in the ^1^H-STD experiments, were prepared by the group at Dublin and their synthesis is reported elsewhere. A stock solution of the fluoro-monosaccharide mixture was prepared in a buffer of 25 mM Tris-HCl, 150 mM NaCl, 4 mM CaCl_2_, pH 8.

### 3.2. Preparation of DC-SIGN Tetramer

The fragment of DNA encoding the full extracellular domain (ECD, residues 70-404) was inserted in a pET15b plasmid and digested with NdeI and BamHI (Thermo Fischer Scientific, Madrid, Spain). The resulting plasmid was transformed on *E. coli* BL21/DE3 competent cells for subsequent overexpression (Sigma-Aldrich). A single colony holding the expression construct was inoculated into 5 mL LB medium containing 100 μg/mL ampicillin and allowed to grow overnight at 37 °C with shaking. The culture was then added to 1 L of LB medium containing ampicillin and grown at 37 °C until exponential phase. The cells were induced with 100 mg/L IPTG and growth continued overnight at 20 °C. The induced culture was harvested by centrifugation at 7500 *g* for 20 min. The pellet was suspended in 10 mL lysis buffer (10 mM Tris-HCl pH 8.0) and the cell suspension was lysed by sonication. Inclusion bodies were isolated via centrifugation at 3000 *g* for 20 min at 4 °C. The insoluble pellet was further solubilized by gentle rotation for 16 h at 4 °C, with 10 mL of pH 8.0 buffer containing 100 mM Tris-HCl, 6 M urea, and 0.01% *v*/*v* 2-mercaptoethanol. The mixture was centrifuged at 100,000 *g* for 2 h at 4 °C, and soluble protein was dialyzed against 2 L of 100 mM Tris-HCl, pH 8.0, 0.01% *v*/*v* 2-mercaptoethanol, 10 mM CaCl_2_, 4 M urea; subsequently against the same buffer with 2 M urea, and finally with no urea. The last dialysis step was done with 100 mM Tris-HCl, pH 8.0, 10 mM CaCl_2_. Then, the precipitate was removed by centrifugation at 100,000 *g* for 30 min at 4 °C. The properly folded DC-SIGN present in the soluble fraction was purified via affinity chromatography using mannose-Sepharose (Sigma-Aldrich), washed in 10 column volumes of loading buffer 20 mM Tris, 150 mM NaCl and eluted in the same buffer containing 10 mM EDTA. Additional purification was performed by size exclusion chromatography using a Superdex 200 column and AKTA liquid chromatography system (GE Healthcare) with a pH 8.0 buffer of 20 mM Tris and 1 mM EDTA. Fractions were concentrated and the buffer was changed to 20 mM Tris, 4 mM CaCl_2,_ and 150 mM NaCl at pH 8.0, then analyzed by 4–12% SDS-PAGE. Protein concentrations were quantified using a NanoDrop UV-Vis spectrophotometer (Thermo Fischer scientific). The identification of the protein was further confirmed by LC-MS. The tetrameric state of the lectin was confirmed by TEM (Jeol JEM-1230, Tokyo, Japan) using negative staining (Figure S8).

### 3.3. ^19^F-Based Screening and Chemical Mapping NMR Experiments

All NMR spectra were recorded on either Bruker AV500 or AV600 NMR spectrometers, equipped with a ^19^F probe (Bruker, Rheinstetten, Germany), 5 mm SEF ^19^F-1H with Z gradient). The ^19^F-NMR transmitter frequencies were 470.56 MHz and 554.56 MHz, respectively. The experiments were carried out at 298 K in the same buffered solution as that of the lectin (20 mM Tris, 4 mM CaCl_2_, and 150 mM NaCl at pH 8.0), as described above. For the ^19^F-NMR signal assignment and sample control, standard coupled and decoupled ^1^H and ^19^F monodimensional spectra were acquired. The ^19^F transmitter frequency offset and spectral width were set to –215 ppm and 50 ppm, respectively, in order to include the range of Larmor frequencies of all species in the mixture. The typical CPMG pulse sequence was used for the relaxation filtered experiments, as follows: [*D*-90*_x_*-(*τ*-180*_y_*-*τ*)*_n_*-acquire], with a recovery delay D=4 s, a free evolution delay τ=2 ms, and n spin-echo loops varying from 2 to 4000, depending on the experiment. The overall time employed corresponds to n times the applied spin echo pulse (n2τ=n4 ms) plus the almost negligible contribution of the refocusing pulse (≈n (15μs)). The T_2_ relaxation times are obtained by fitting the observed data points to the exponential decay curve:

(2) $$I\left(t\right)=I_{0}e^{-t/T_{2}}=I_{0}e^{-n2\tau /T_{2}}$$

where, I(t) stands for the intensity of a particular peak at time t, $I_{0}$ is its intensity at t=0, and $T_{2}$ is the transversal relaxation time.

For the ^19^F-NMR relaxation filter experiments, an aliquot of the library diluted to 1 mM (i.e., for each sugar type the concentration of each anomer is: [α]+[β]≅1 mM) was used. After measure transversal relaxation times of the species in the free state, T_2,free_, several additions of DC-SIGN tetramer directly into the NMR tube were carried out and the observed transversal relaxation times, T_2,obs_, computed. The range of ligand/protein ratios covered in the experiments varied from 23 to 235. The employed lectin tetramer (ECD) concentrations varied from 16 to 104 µM, while the ligand concentrations, varied from 600 to 890 µM.

To reduce the likelihood of observing nonspecific binding, low ligand/receptor molar ratios were explored. Moreover, relaxation filter experiments were repeated after addition of a known competitor, Manα1-3Manα1-6-Man (Carbosynth), to the F-sugar/lectin mixtures (Figure S7).

Control experiments varying the 180° pulse repetition frequency using different *τ* delays between 0.1 and 20 ms for 4F-Man in the presence of DC-SIGN showed the presence of chemical exchange. This fact hampers the estimation of quantitative binding affinities from the observed T_2_ values [24,25]. In any case, in order to minimize this effect short *τ* delays (2 ms) were used with the monosaccharide mixture. Given the close chemical nature of the ligands, the exchange contribution was expected to be relatively similar for these molecules, allowing us to make justifiable qualitative conclusions regarding binders versus non-binders.

### 3.4. ^1^H-STD NMR Experiments

^1^H-STD NMR experiments [1] were acquired on a Bruker AV600 NMR spectrometer at 298 K for a sample containing α-1-Met-4-F-Man (5 mM) and DC-SIGN CRD (65 uM), corresponding to a 77/1 ligand/protein molar ratio. A deuterated buffer for the STD experiments was used, similar to that described for the CPMG experiments: 20 mM Tris-d_11_, 4 mM CaCl_2_, and 150 mM NaCl at pH 8.0 in D_2_O. The on-resonance irradiation was set at 0.81 ppm to selectively saturate the aliphatic region of the lectin, while the off-resonance frequency was set at 60 ppm. There were 1024 scans recorded in each experiment, using 6 s of recovery delay, with a spin-lock relaxation filter of 20 ms to reduce the receptor background signals. For selective protein saturation, the standard Bruker gradient shape file was used, SMSQ10.100, which is rectangular in shape with smoothed edges to give a gradient integral of 90% of a square pulse. The STD build-up curve for each proton was computed by conducting the experiment at four saturation times, from 0.5 to 3.5 s. The STD experimental data was fitted to the mono-exponential equation [26]:

(3) $$STD\left(t\right)=STD^{max}\left(1-e^{\left(-k_{sat}*t\right)}\right)$$

where, STD(t) is the STD signal intensity of a particular proton at saturation time t, $STD^{max}$ stands for the maximum value of the STD intensity curve at long saturation times, and $k_{sat}$ refers to the experimental saturation rate constant. To avoid T_1_ bias, the STD contribution of each proton was estimated from the slope of the STD build-up curve at saturation time zero (Table S10, Figure S6), and a relative scale of STD (fit) values referenced to proton H4, which displays the highest STD for epitope mapping discussion. The T_1_ relaxation time of each proton was further estimated employing the inversion recovery pulse sequence (Table S10). Control experiments were also performed in the presence of EDTA to sequester the Ca^2+^ ion. As expected, no STD was detected under these conditions.

### 3.5. Molecular Dynamics Simulations

The lectin structure used in all the simulations was derived from the high-resolution crystal structure of DC-SIGN CRD bound to LNFP III (PDB code 1SL5) [17]. The structure contained three Ca^2+^ ions, one of them directly bound to the ligand. The spatial coordinates of all the sugar atoms employed in the computational studies were generated as follows:

1. For the simulations conducted using the sugar geometries of the crystal:
  - The starting structure of α-Fuc from the original 1SL5 ligand was used, and the remaining parts of LNFP III were removed;
  - The α-Man starting geometry was built from another DC-SIGN crystal in complex with a high-mannose derivative (PDB code 2IT5, using the geometry of the sugar bound in the major orientation) [27], removing the remaining residues.
2. For the simulations conducted using the geometry of the proposed binding poses A and B:
  - The OH-2/OH-3 groups of β-4-F-Man were manually superimposed onto the OH-4/OH-3 pair of L-Fuc from the original crystal structure 1SL5 to create the model for binding pose A. Binding pose B was generated by superimposing the OH-2/OH-3 groups of β-4-F-Man to the OH-4/OH-3 pair of Man from the structure 2IT5;
  - The α-Man starting structure was built as in the case of β-4-F-Man.

MD simulations were conducted using version 16 of the Amber molecular dynamics software package [28]. All simulations were run in explicit TIP3P water [29]. The ff14SB [30] and GAFF [31] force fields were employed for the description of the protein and monosaccharides (both natural and fluorinated), respectively. In addition, the MD simulations with the natural sugars (nonfluorinated) were repeated using the GLYCAM [32] force field, as a control for those simulations run using the GAFF parameters for the sugar. The Li/Merz ion parameters for the ions Ca^2+^ and Cl^-^ (necessary to neutralize the charge of the system) in TIP3P water (12–6 normal usage set) were employed [33].

A general protocol consisting of two minimization stages, heating of the system from 0 to 300 K, density equilibration for a total of 2 ns and production dynamics in the NPT ensemble at 300 K/1 bar was employed in every case. The long-range electrostatic interactions were treated using the particle mesh Ewald summation. In order to maintain a constant temperature, the Langevin thermostat with a collision frequency of 1 ps was used. Simulation times from 20 to 50 ns were used for the study of the 4-F-Man/DC-SIGN complexes, and 100 to 200 ns for the natural sugar complexes.

## 4. Conclusions

The screening and chemical mapping strategy was based on simple ^19^F-NMR relaxation filter experiments in order to assess that monosaccharides Fuc, Man, Glc, and Gal bind to DC-SIGN with decreasing affinity, the latter showing extremely weak, but still detectable binding. Moreover, a new binding mode for Man moieties was detected and described, which involves the direct coordination of DC-SIGN Ca^2+^ by OH-2 and OH-3 groups at the sugar recognition site, by employing a combination of ^1^H-STD experiments and MD simulations. Our results suggest that two possible binding modes, via O3/O4-Ca^2+^ and via O2/O3-Ca^2+^, may coexist for the natural sugar. In fact, the detailed analysis of the interaction network formed in both cases indicates that more favorable contacts are present in the new pose, resembling those found on Fuc units. The biological relevance of these findings is still to be demonstrated, but could open new avenues for the design and synthesis of novel DC-SIGN inhibitors.

## Figures and Tables

**Table 1:** Fluorinated monosaccharides that display effective binding to DC-SIGN and observed averaged decrease in T_2_. These values are qualitatively related with the relative binding affinity: Fuc moieties interact better, followed by Man [13]. The Gal moieties displayed a small change in the observed averaged decrease in T_2,free_, highlighting that they are rather poor binders of DC-SIGN [13]. The average T_2,free_ was calculated for all the species depending on the particular sugar. The average T_2_ decay % was computed for those species that exhibited a T_2_ decay over 40%.

Sugar	T_2,free_ Average	% T_2,decrease_ Average	Binding Molecules
Fuc	2.0	90	(α,β)-2-F-Fuc
Man	1.3	70	(α,β)-2-,4-,6-F-Man
Glc	1.3	60	(α,β)-2-,6-F-Glc
Gal	1.8	51	(α,β)-2-F-Gal

## Supplementary Materials

The following are available online. Figure S1. The complete MD simulation of the complex between DC-SIGN/D-Man starting from binding pose A shows the sugar moiety switching its position from the starting geometry. Figure S2. Populations of the different HB found along the MD simulation (187 ns) of the D-Man/DC-SIGN complex using binding pose A as starting geometry, which switches to the X-ray crystallographic pose. Figure S3. Populations of the different HB found along the MD simulation of the D-Man/DC-SIGN, 4-F-Man/DC-SIGN, and L-Fuc/DC-SIGN complexes using the corresponding binding poses A as starting geometries. Figure S4. Populations of the different HBs found along the MD simulation of the D-Man/DC-SIGN and 4-F-Man/DC-SIGN complexes using binding pose B as starting geometry. Figure S5. Superimposition of two snapshots taken from the MD simulations carried out for D-Man and 4-F-Man (yellow) bound to DC-SIGN using pose B. Figure S6. STD build-up curves for α-OMe-4-F-Man as a function of the saturation time. Figure S7. Superimposition of ^19^F-NMR relaxation filter spectra in absence (green) and presence (red and blue) of a competing molecule (Manα1-3Manα1-6-Man) to assess the specific binding of some fluorinated monosaccharides to DC-SIGN. Figure S8. Electron microscopy pictures using negative staining. Table S1: Transverse relaxation times deduced for the fluorine-containing monosaccharide library using the CPMG spin echo pulse sequence. Table S2. Main interactions found on the MD simulations for L-Fuc bound to DC-SIGN in the X-ray crystal structure. Table S3. Main interactions found on the MD simulations performed for D-Man bound to DC-SIGN in the X-ray crystal structure. Table S4. Major stabilizing interactions found on the first part (ca. 112 ns) of the MD simulation performed for D-Man in binding pose A and DC-SIGN. Table S5. Major stabilizing interactions found on the second part (ca. 113–185 ns) of the MD simulation performed for DC-SIGN and D-Man in binding pose A as the starting configuration. Table S6. Main interactions found on the complete MD simulations of the complex between DC-SIGN/D-Man starting from binding pose A. Table S7. Key interactions in the MD simulations performed for the complex between D-Man and DC-SIGN using binding pose B as starting geometry. Table S8. Main interactions in the MD simulations performed for the complex between 4-F-Man and DC-SIGN using binding pose A as starting geometry. Table S9. Main interactions In the MD simulations performed for the complex between 4-F-Man and DC-SIGN using binding pose B as starting geometry. Table S10. Initial STD slope (t = 0) for each proton of the 4F-Man OMe derivative calculated from the fitted exponential equation.
