# Supplementary material for: Unraveling Sugar Binding Modes to DC-SIGN by Employing Fluorinated Carbohydrates
Source: Molecules. 2019 Jun 25;24(12):2337. doi: 10.3390/molecules24122337 (PMC6631030; doi:10.3390/molecules24122337)
Supplement: Supplementary file 1 [file molecules-24-02337-s001.pdf]

SUPPORTING INFORMATION

Tables

Table 1. Transverse relaxation times deduced for the fluorine-containing monosaccharide library using the CPMG spin echo pulse sequence.

Peak	δ (ppm)	Assigment	T_2 free (s)
1	-195.041	3F-β-Glc	1.46
2	-198.140	4F-α-Glc	1.59
3	-198.675	3F-β-Man	1.58
4	-199.151	3F-β-Gal	1.74
5	-199.273	2F-β-Glc	1.61
6	-199.412	2F-α-Glc	1.90
7	-199.939	3F-α-Glc	1.42
8	-200.153	4F-β-Glc	1.56
9	-203.250	3F-α-Gal	1.77
10	-203.870	3F-α-Man	1.30
11	-204.747	2F-α-Man	1.68
12	-204.802	4F-α-Man	0.70
13	-207.385	2F-β-Gal	1.66
14	-207.542	2F-α-Gal	1.94
15	-207.730	2F-β-Fuc	1.89
16	-207.974	2F-α-Fuc	2.16
17	-208.374	4F-β-Man	1.54
18	-217.479	4F-β-Gal	1.57
19	-220.024	4F-α-Gal	1.73
20	-223.158	2F-β-Man	1.80
21	-229.641	6F-β-Gal	1.40
22	-229.779	6F-α-Gal	1.40
23	-233.625	6F-β-Man	0.99
24	-234.449	6F-α-Man	0.83
25	-234.773	6F-β-Glc	0.92
26	-235.497	6F-α-Glc	0.90

Table 2. Main interactions found on the MD simulations for L-Fuc bound to DC-SIGN in the X-Ray crystal structure. Fraction is referred to the number of frames in which the complex is fully associated. In this case, ca. 100 % of the total simulation time (100 ns).

	**Donor**	**Aceptor**	**Avg Distance (A)**	**Fraction**
Hydrogen Bond	α-Fuc HO4	Glu347 O	2.6	1
	α-Fuc HO2	Glu354 O	2.6	1
	α-Fuc HO3	Glu354 O	2.7	0.89
	Asn365 NH	α-Fuc O3	3.2	0.81
	Asn349 NH	α-Fuc O4	3	0.70
Charge-dipole	α-Fuc O3	Ca2+	2.65	0.99
	α-Fuc O4	Ca2+	2.6	1
	**Atom1**	**Atom2**	**Avg Distance (A)**	**Fraction**
Van der Waals	Val351 H	α-Fuc H2	2.4	0.90

Table S3. Main interactions found on the MD simulations performed for D-Man bound to DC-SIGN in the X-Ray crystal structure. Fraction is referred to the number of frames in which the complex is fully associated. In this case, ca. 100 % of the total simulation time (100 ns).

	Donor	Aceptor	Avg Distance (A)	Fraction
Hydrogen Bond	β-Man HO4	Glu354 O	2.65	0.99
	Asn365 NH	β-Man O4	3.1	0.96
	β-Man HO3	Glu347 O	2.65	0.88
	Asn349 NH	β-Man O3	3.1	0.50
Charge-dipole	β-Man O3	Ca2+	2.6	1
	β-Man O4	Ca2+	2.65	0.96

Table S4. Major stabilizing interactions found on the first part (ca. 112 ns) of the MD simulation performed for D-Man in binding pose A and DC-SIGN. Fraction is referred to the number of frames in which the complex is fully associated before the switch in the binding pose occurs. In this case, ca. 56 % of the simulation time (200 ns).

	**Donor**	**Aceptor**	**Avg Distance (A)**	**Fraction**
Hydrogen Bond	β-Man HO4	Glu354 O	2.7	0.95
	β-Man HO2	Glu347 O	2.6	0.94
	β-Man HO3	Glu354 O	2.7	0.91
	Asn365 NH	β-Man O3	3.1	0.87
	Asn349 NH	β-Man O2	3.1	0.51
Charge-dipole	β-Man O2	Ca2+	2.6	1
	β-Man O3	Ca2+	2.6	0.96
	**Atom1**	**Atom2**	**Avg Distance (A)**	**Fraction**
Van der Waals	Val351 H	β-Man H4	2.8	0.86

Table S5. Major stabilizing interactions found on the second part (ca. 113-185 ns) of the MD simulation performed for DC-SIGN and D-Man in binding pose A as the starting configuration. These interactions are similar to those found on the X-Ray structure of D-Man at DC-SIGN's binding site (Table S3). Fraction is referred to the number of frames in which the complex is fully associated after the switch in the binding pose (113-185 ns). In this case, ca. 36 % of the simulation time (200 ns).

	**Donor**	**Aceptor**	**Avg Distance (A)**	**Fraction**
Hydrogen Bond	β-Man HO4	Glu354 O	2.7	1
	Asn365 NH	β-Man O4	3.0	0.98
	β-Man HO3	Glu347 O	2.7	0.91
	Asn349 NH	β-Man O3	3.1	0.17
Charge-dipole	β-Man O3	Ca2+	2.6	1
	β-Man O4	Ca2+	2.7	1

Table S6. Main interactions found on the complete MD simulations of the complex between DC-SIGN/D-Man starting from binding pose A. Fraction is referred to the number of frames in which the complex is fully associated. In this case, ca. 93.5 % of the total simulation time (187 ns). The relative populations of both binding modes in this MD simulation can be extracted from the relative fractions of the oxygen-calcium interactions.

Type of interaction	Donor	Aceptor	Avg Distance (A)	Fraction
Hydrogen Bond	β-Man HO2	Glu354 O	2.7	1
	β-Man HO2	Glu347 O	2.7	0.59
	β-Man HO3	Glu354 O	2.7	0.57
	Asn365 NH	β-Man O3	3.1	0.54
	Asn365 NH	β-Man O4	3	0.40
	β-Man HO3	Glu347 O	2.7	0.39
Charge-dipole	β-Man O2	Ca2+	2.6	0.62
	β-Man O3	Ca2+	2.6	0.99
	β-Man O4	Ca2+	2.7	0.4
	**Atom1**	**Atom2**	**Avg Distance (A)**	**Fraction**
Van der Waals	Val351 H	β-Man H4	2.8	0.57

Table S7. Key interactions in the MD simulations performed for the complex between D-Man and DC-SIGN using binding pose B as starting geometry. Fraction is referred to the number of frames in which the complex is fully associated. In this case, ca. 100 % of the total simulation time (200 ns).

	**Donor**	**Aceptor**	**Avg Distance (A)**	**Fraction**
Hydrogen Bond	Man HO4	Glu347 O	2.7	0.98
	Asn365 NH	Man O2	3.1	0.95
	Man HO2	Glu354 O	2.7	0.91
	Asn349 NH	Man O3	3.2	0.36
	Man HO3	Glu347 O	2.8	0.28
Charge-dipole	β-Man O2	Ca^{2+}	2.6	1
	β-Man O3	Ca^{2+}	2.7	1

Table S8. Main interactions in the MD simulations performed for the complex between 4-F-Man and DC-SIGN using binding pose A as starting geometry. Fraction is referred to the number of frames in which the complex is fully associated. In this case, ca. 15 % of the total simulation time (100 ns).

	Donor	Aceptor	Avg Distance (A)	Fraction
Hydrogen Bond	β-4F-Man HO2	Glu347 O	2.6	1
	β-4F-Man HO3	Glu354 O	2.7	0.98
	Asn365 NH	β-4F-Man O3	3.2	0.71
	Asn349 NH	β-4F-Man O2	3	0.55
Charge-dipole	β-4F-Man O2	Ca2+	2.6	1
	β-4F-Man O3	Ca2+	2.6	1
	**Atom1**	**Atom2**	**Avg Distance (A)**	**Fraction**
Van der Waals	Val351 H	β-4F-Man H4	2.7	0.94

Table S9. Main interactions In the MD simulations performed for the complex between 4-F-Man and DC-SIGN using binding pose B as starting geometry. Fraction is referred to the number of frames in which the complex is fully associated. In this case, ca. 40 % of the total simulation time (100 ns).

	**Donor**	**Aceptor**	**Avg Distance (A)**	**Fraction**
Hydrogen Bond	Asn365 NH	β-4F-Man O2	3.05	0.99
	β-4F-Man HO3	Glu347 O	2.7	0.94
	β-4F-Man HO2	Glu354 O	2.8	0.75
	Ser360 OH	β-4F-Man O6	3.0	0.23
Charge-dipole	β-4F-Man O2	Ca2+	2.63	0.98
	β-4F-Man O3	Ca2+	2.58	1

Table S10. Initial STD slope (t=0) for each proton of the 4F-ManαOMe derivative calculated from the fitted exponential equation. % STD (fit) values are normalized with respect to H4, set to 100%. As expected, T_1 relaxation values for H6s are considerable lower than those exhibited by the other protons, highlighting the importance of using initial slopes (at t_{sat}=0) to avoid misinterpretation of ligand-receptor proton distances. H5 and H6 build-up curves practically converge at 3.5 of protein saturation, whereas H2 and H4 are still growing at that point.

	**STDmax*Ksat*10-3**	**% STD (fit)**	**T1 (s)**
**H4**	4.323	100.00	2.24
**H3**	1.987	52.07	2.26
**H2**	1.662	40.25	2.86
**H6**	2.808	64.94	0.94
**H5**	2.590	59.90	-

Figures

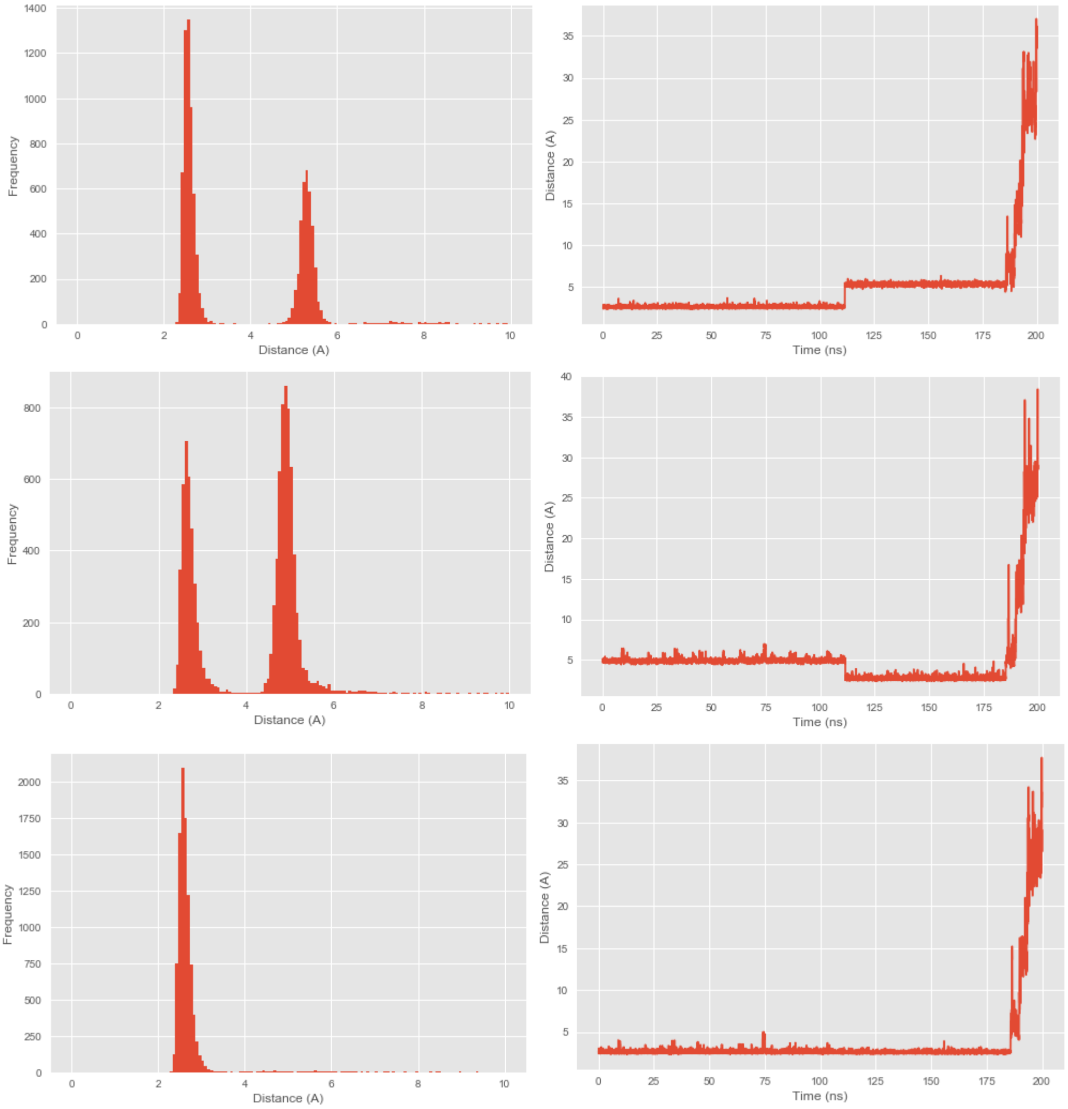

Figure S1. The complete MD simulation of the complex between DC-SIGN/D-Man starting from binding pose A shows the sugar moiety switching its position from the starting geometry. The distance between Man O2, O3 and O4 groups and the Ca2+ ion is shown. Top) frequency and trajectory of the O2-Ca2+ distance. Middle) frequency and trajectory of the O4-Ca2+ distance. Bottom) frequency and trajectory of the O3-Ca2+ distance. The positional switch between binding pose A (O2/O3 attached to the Ca2+ ion) and the X-Ray binding pose (O3/O4 attached to the Ca2+) ion takes place at ca. 113 ns.

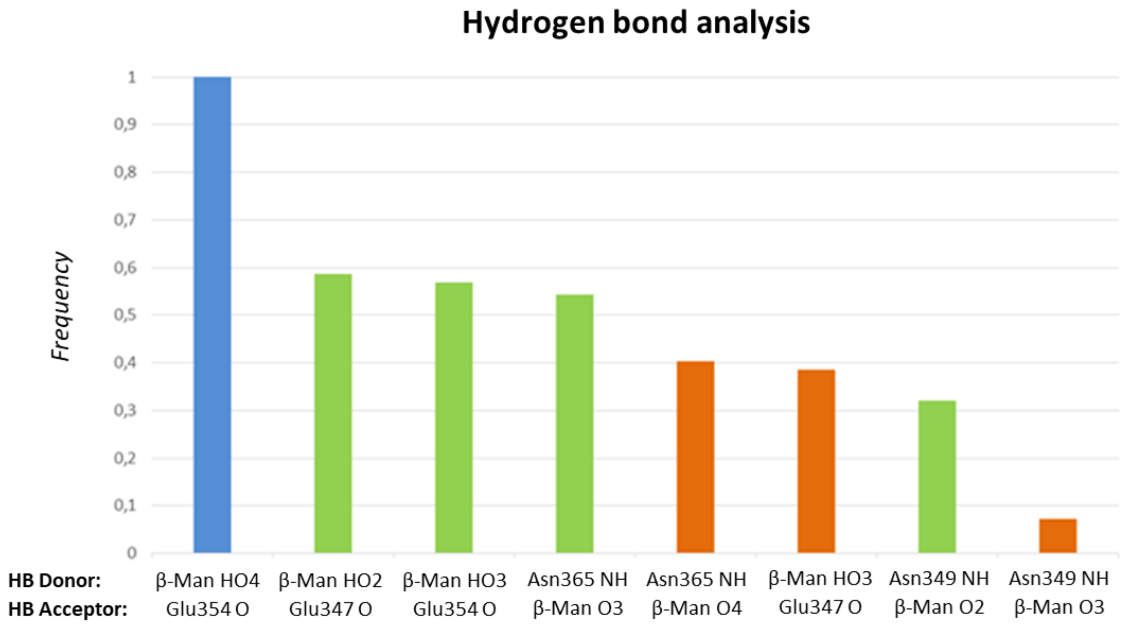

Figure S2. Populations of the different HB found along the MD simulation (187 ns) of the D-Man/DC-SIGN complex using binding pose A as starting geometry, which switches to the X-Ray crystallographic pose. The blue bar refers to the HB shared in both binding modes. The green and orange are characteristic for Man in binding pose A and in the X-Ray structure, respectively.

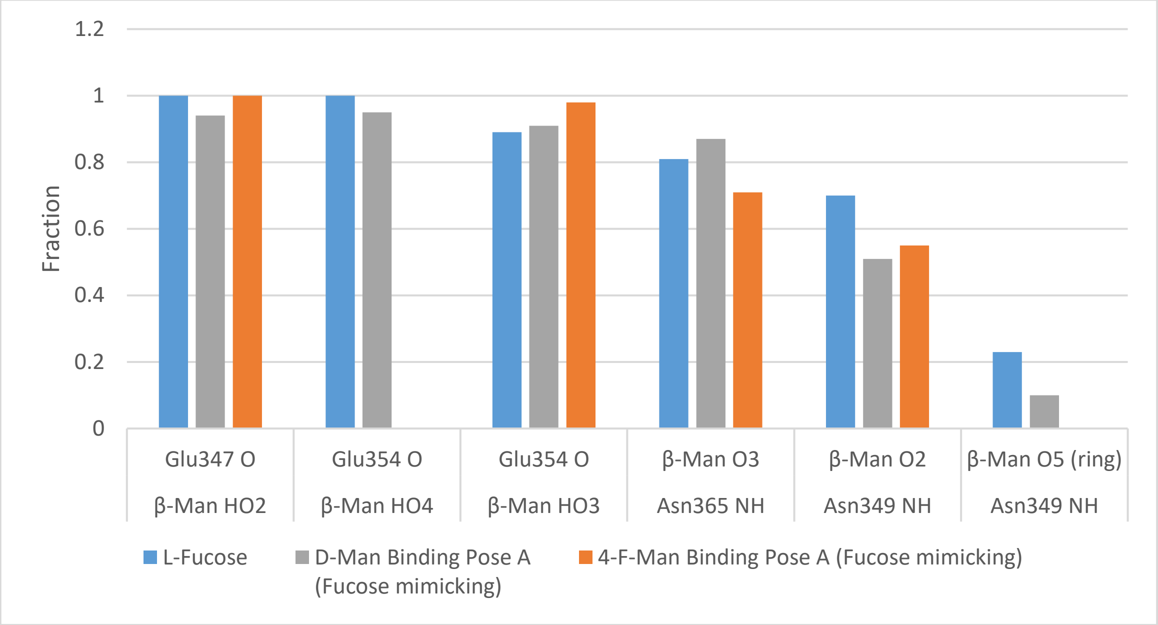

Figure S3. Populations of the different HB found along the MD simulation of the D-Man/DC-SIGN, 4-F-Man/DC-SIGN, and L-Fuc/DC-SIGN complexes using the corresponding binding poses A as starting geometries. For D-Man and L-Fuc, the data refer to the complete 100 ns simulation time, while for 4-F-Man, it is referred to the time the complex remain fully associated, 15 ns.

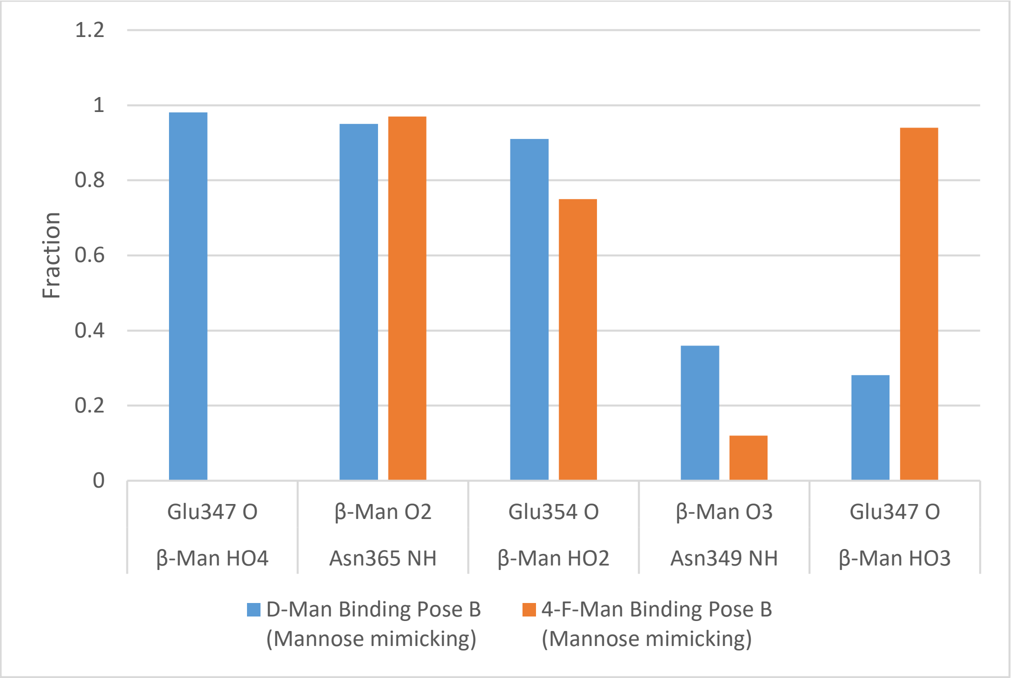

Figure S4. Populations of the different HBs found along the MD simulation of the D-Man/DC-SIGN and 4-F-Man/DC-SIGN complexes using binding pose B as starting geometry. The role of Man HO3 as donor is drastically enhanced in the 4-F-Man analogue with respect to Man, obviously due to the lack of HO4 in the fluorinated compound. For D-Man, the data refer to the complete 200 ns MD simulation time, while for 4-F-Man, it is referred to the time the complex remain fully associated, 50 ns.

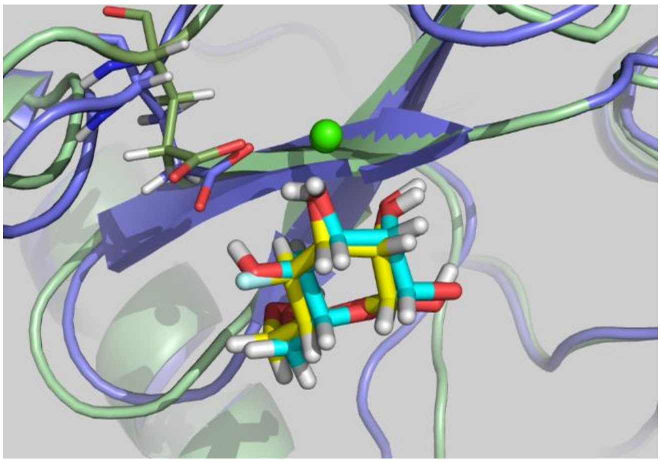

Figure S5. Superimposition of two snapshots taken from the MD simulations carried out for D-Man and 4-F-Man (yellow) bound to DC-SIGN using pose B. The green backbone corresponds to the complex with 4-F-Man. The key difference involves the role of Glu347, which establishes HB interactions with OH4 (D-Man) and with OH3 (4-F-Man).

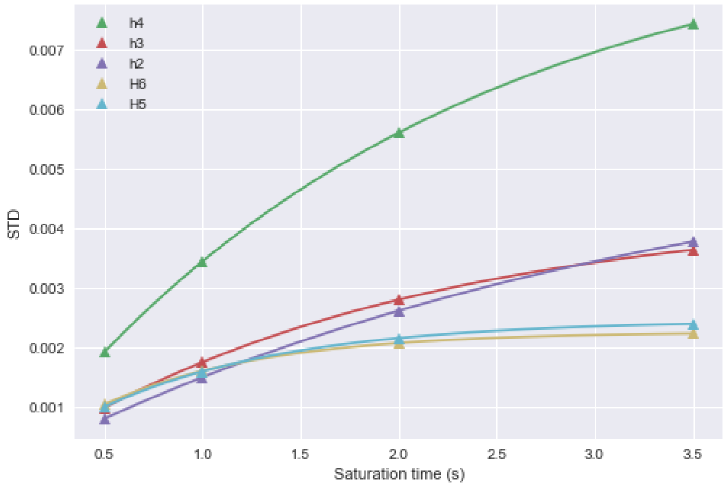

Figure S6. STD build-up curves for 4F-ManαOMe as a function of the saturation time. The STD^{max} value and the saturation rate constant k_{sat} were derived by least-squares fitting of the experimental data (triangle) to the monoexponential function $STD = STD^{max}(1 - e^{(-k_{sat}*t)})$.

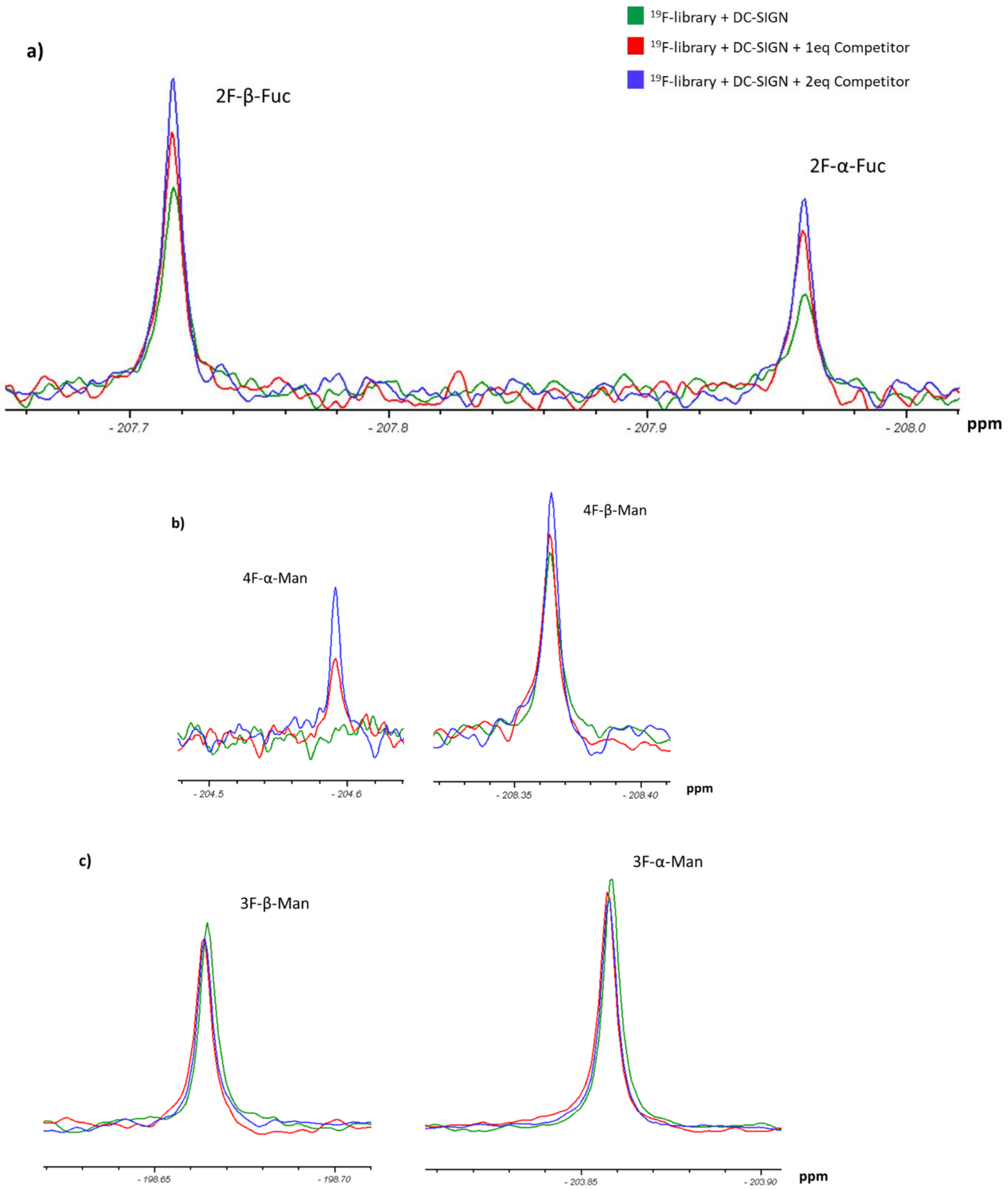

Figure S7. Superimposition of ^{19}F-NMR relaxation filter spectra in absence (green) and presence (red and blue) of a competing molecule (Manα1-3Manα1-6-Man) to assess the specific binding of some fluorinated monosaccharides to DC-SIGN. The T_2 relaxation filter duration was 241 ms. The equivalents of the competitor are with respect to the concentration of the fluorinated monosaccharide mixture (for each sugar type the concentration of each anomer is: $[\alpha] + [\beta] \cong 0.55\ mM$). The close-ups corresponds to the binders α- and β-2-F-Fuc (a)), α- and β-4-F-Man (b)), and the non-binders α- and β-3-F-Man.

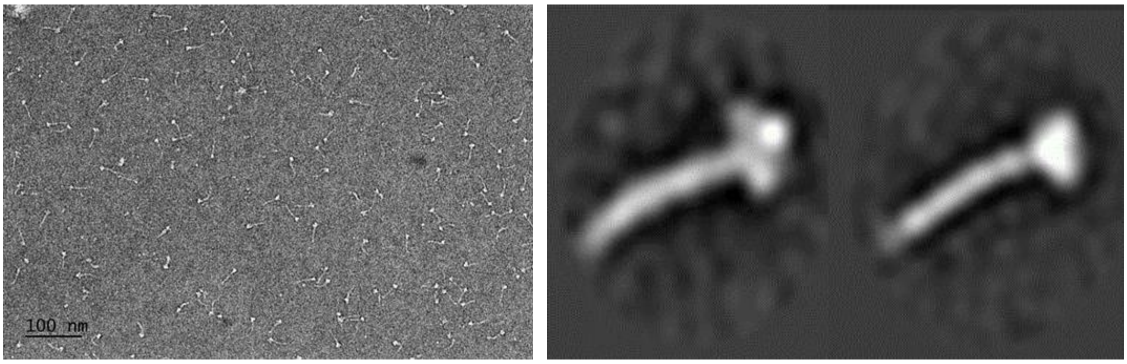

Figure S8. Electron microscopy pictures using negative staining. Representative areas of digital micrographs are shown at the left. Selected class averages resulting from 2D reference-free alignment at the right. Model exhibit a tail with a length of 245–250 Å and a diameter of 25–30 Å, whereas the head has a length of 70 –75 Å and transverse dimensions in the range of 50 to 80 Å.
